# Comparative performance analysis of U-Net and DeepLabV3+ for semantic segmentation in traffic environments

**DOI:** 10.1038/s41598-026-46740-2

**Published:** 2026-04-01

**Authors:** Ramyashree, S. Rai Utsavi, S. Raghavendra, B. N. Anoop, P. S. Venugopala

**Affiliations:** 1 Manipal Institute of Technology, Manipal Academy of Higher Education, Manipal, Karnataka 576104 India; 2 Department of Artificial Intelligence and Data Science, NMAM Institute of Technology, Nitte (Deemed to be University), Karkala, Karnataka 574110 India

**Keywords:** Engineering, Mathematics and computing

## Abstract

Computer vision is an important field of artificial intelligence that enables machines to interpret and understand visual information from images. It is the basis of automated visual understanding in smart systems. Monitoring of streets, recognizing different vehicles and pedestrians, and comprehending the ever-changing traffic conditions for making right decisions are some of the features of modern intelligent traffic systems. It is indispensable for a computer to visually recognize the semantic segmentation (SS) of a scene, as it maps each and every pixel of the image to the categories like roads, vehicles, pedestrians, traffic lights, etc. However, existing traffic scene segmentation models often perform poorly under challenging conditions such as low lighting, blur, noise, and low resolution. These limitations limit the robustness of these methods. They can be a major obstacle to the development of autonomous vehicle technologies because they reduce the ability of perception systems to recognize a wide range of situations. This work proposes a new method for processing poor quality traffic images by sequentially applying super-resolution (SR), semantic segmentation (SS), and object detection with YOLOv8x. The SR module transforms degraded inputs, while U-Net and DeepLabV3+ are exploited for accurate pixel-level segmentation mask generation. Besides, YOLOv8x provides the precise object detection, which is really one of the critical steps for the avoidance of the errors in the crowded and complicated TSs. YOLOv8x uses bounding boxes to check segmentation and raise mAP. U-Net delivers PSNR of 41.93 dB, SSIM of 0.997, mIoU of 0. 750, and mAP of 0.950, whereas DeepLabV3+ yields PSNR of 46.03 dB, SSIM of 0.938, mIoU of 0.819, and mAP of 0.937.

## Introduction

Semantic segmentation is indeed one of the very difficult problems in computer vision that computer vision researchers have been working on. It means to assign a label to each pixel of an image, describing a class that the pixel belongs to, which can be of utmost importance in such areas as medical imaging, remote sensing, and autonomous vehicles^1–3^. Semantics is a very important aspect to know. Low resolution, low contrast, different kinds of lighting, and background noise can all have a negative impact on the accuracy of segmentation, especially when there are complex scenes like organ regions or densely populated urban areas. These factors are the reasons of blurred edges, wrong label assignment and missed detection of small and overlapping objects^4–6^. Traditional segmentation models usually depend on perfect data and may not work properly in real-world scenarios where there could be noise and other variations. To overcome such issues, recent developments have begun to present image enhancement as an essential preprocessing step before segmentation^5,7^. Methods like contrast-limited adaptive histogram equalization (CLAHE), denoising filters, and super-resolution upscaling are commonly used to enhance input clarity and maintain texture features^8^. Most traditional models of traffic-scene segmentation fail to perform well when the images are at low light, have motion blur, noise, or low resolution-all common conditions in natural driving. In such conditions, several of these existing models either cannot detect objects accurately or clearly delineate their boundaries, which significantly diminishes the quality of segmentation^9^. This paper proposes an integrated pipeline that combines super-resolution preprocessing, object detection using YOLOv8, and semantic segmentation using DeepLabV3+/U-Net. It enhances the visual quality of the input image, improves the alignment between the detected objects and their segmented regions, and enhances robustness under adverse conditions.We introduce a method that is less error-prone for complicated and deteriorated traffic scenes through the integration of detection, enhancement, and segmentation processes into a single framework. Refined images carry more differentiating features, thus, segmentation models can recognize patterns at a higher level. The effect of these refinements on the accuracy and trustworthiness of the semantic prediction can be very significant when they are combined with state-of-the-art deep learning architectures, in particular, in situations with noise or limited data.Deep learning models like UNet and DeepLabV3+ have been quite effective in these scenarios. These models offer different strengths which complement each other: UNet is especially capable of retaining spatial accuracy and boundary detail through encoder-decoder skip connections, and DeepLabV3+ enrich the semantic content by using Atrous Spatial Pyramid Pooling (ASPP) to get the context from different scales^1,9,10^. They all, however, have only one failure point–UNet might be missing the general context, and DeepLabV3+ could hardly distinguish the faint boundary lines. As shown in flowchart image upscaling is the first step which enhances the input through resolution increase, better contrast, and feature richness. After visually enhancing the image, two parallel semantic segmentation pipelines U-Net and DeepLabV3+ process it. Both of them produce their segmentation maps separately. Two models are used for their different qualities: U-Net gives a local precision with fine edge details whereas DeepLabV3+ achieves a wider semantic context through the use of dilated convolutions. Then a refinement U-Net is employed helping to improve spatial consistencies and to correct minor segmentation errors.

**Fig. 1:**
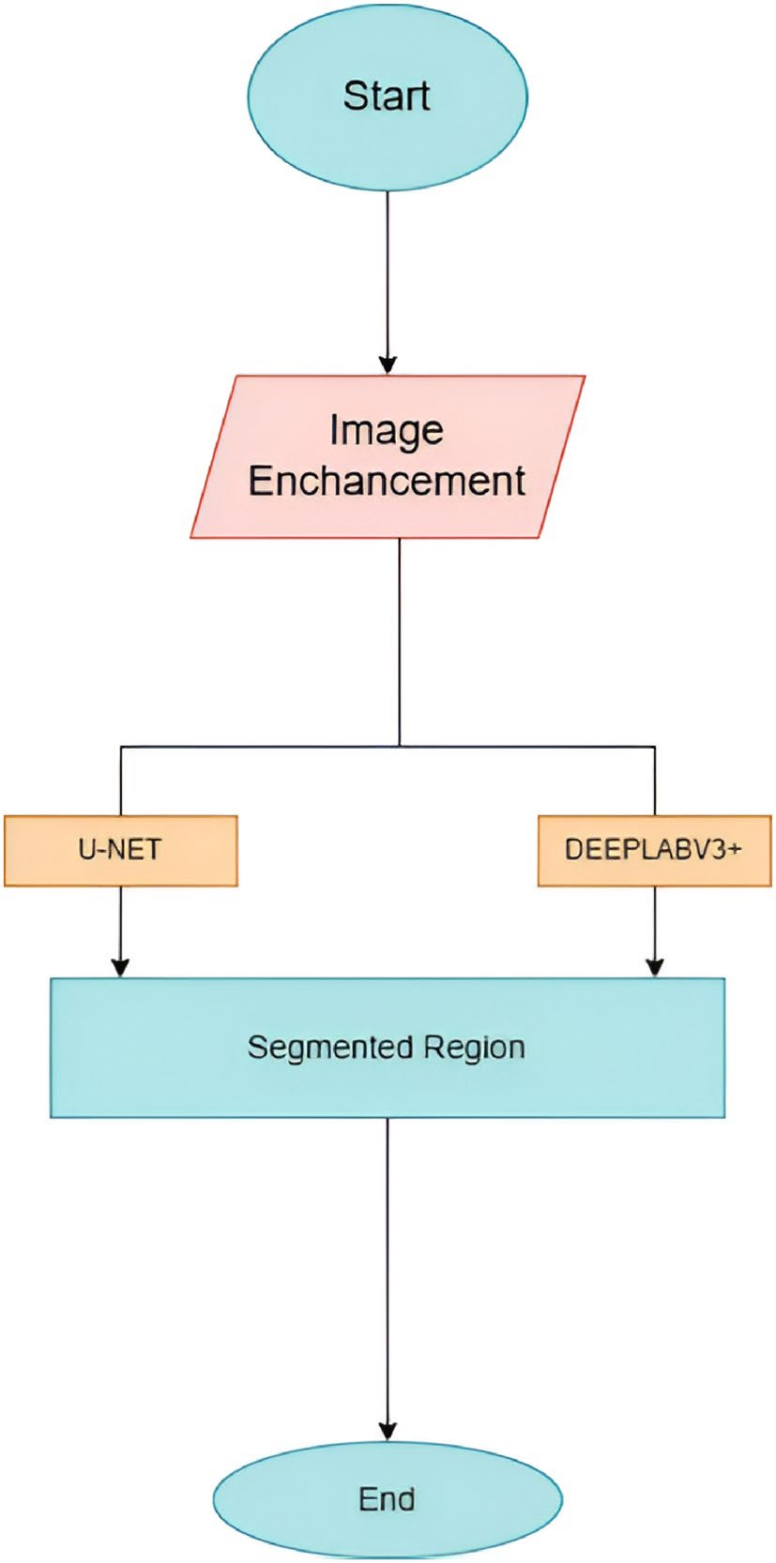
Integrated framework architecture.

The ultimate result is one segmentation map that has been obtained by merging each model’s output. Such a map could serve as a source for subsequent processing, extraction of features, classification of regions, or making a decision in medical imaging or object recognition, to name a few. The refined segmentation maps not only elevate the machine’s perception but also pave the way for the sophisticated processing, which is of great use, inter alia, in the medical sector, earth observation, and smart automation^6,7^. This research showcases a segmentation–detection pipeline predominantly aimed at intricately detailed traffic scenes. Initially, the work is centered around a super-resolution method that is intended to lessen the impact of difficult image conditions like low light, noise, motion blur, and low resolution. After that, the improved result is fed to semantic segmentation with U-Net and DeepLabV3+, thus giving the system not only the detailed structural information of the scene but also the wider semantic context. The final step uses YOLOv8 to determine whether the segmented areas are indeed the real objects in the scene. The system can continue its operations smoothly and faultlessly even in challenging and degraded traffic situations, which is one of the reasons why it is more reliable and robust than ever. The full semantic segmentation framework is illustrated in Fig. 1. The procedure is illustrated by the figure. Figure 1 illustrates the complete semantic segmentation pipeline. Initially, the input picture undergoes enhancement to raise its resolution and visual quality. Next, the enhanced image will be simultaneously fed to U-Net and DeepLabV3+ for segmentation. This is how it is possible to gain better and more trustworthy segmentation outcomes?. While U-Net is more about preserving spatial detail and boundary accuracy, DeepLabV3+ is able to capture semantic context by its multi-scale feature extraction. Both models are separate and thus, they produce segmentation results of their own. This paper is the first to present a novel and integrated coupling segmentation framework that links super-resolution with U-Net and DeepLabV3+ not only for spatial detail recovery but also for mask prediction enhancement in low-resolution or visually degraded environments. When structural information is enhanced prior to segmentation, the pipeline is able to carry out pixel-wise classification that is more accurate in complex traffic scenes. In addition, the study goes to great lengths to refine the parameters that affect the performance of the system such as super-resolution scaling factors, learning rates and segmentation hyper parameters to achieve stable and balanced performance in both the reconstruction and segmentation tasks. The experimental results show that there are continuous improvements both at the pixel level (PSNR, SSIM, MSE, mIoU) and at the object level (mAP) thus, confirming the benefits of the integrated design. While this research has no new architectural contributions, the main contribution of this research is the coordination of super-resolution (ESRGAN) with the established segmentation models (U-Net and DeepLabV3+) to form a single enhancement–segmentation pipeline. This fusion increases the recovery of features and the quality of masks in images that are of low resolution or have been degraded, thus making the approach extremely valuable in fields like vehicle and traffic analysis. Therefore, it is a system-level innovation and a domain-optimized performance solution that does not conflict with current state-of-the-art methods but rather complements them without the need for new architectural designs. In addition, several qualitative experiments, which were carried out under various environmental conditions, e.g. daytime and nighttime illumination, light rain, fog-induced low contrast, and several camera viewpoints, attest to the real-world robustness of the proposed system. All the references have been verified and reordered to make sure that they are in the correct order and to improve the academic readability of the paper.

Their outputs are assessed individually to see the behavior under different visual conditions.Our work major contributions are:

1. Perform, analyze and compare the performance of deep learning models, i.e. DeepLabV3+ and U-Net which are used for semantic segmentation of traffic images.
2. Using the segmented images generated by the models as ground truth for the evaluation of object detection accuracy, thereby allowing the proper classification of objects and the making of right decisions.
3. To evaluate the performance of the models through various metrics.

The record is divided into multiple segments. “Related work” section offers an in-depth description of the Literature Review, concentrating on the previously published studies for semantic segmentation and object detection. “Methodology” and “Experiment setup and result analysis” sections detail the proposed hybrid pipeline, which integrates DeepLabV3+ and U-Net for semantic segmentation and YOLOv8x for object detection, followed by a complete analysis of performance metrics including PSNR, SSIM, mIoU, and mAP. Finally,  “Conclusion and future work” section concludes the paper by summarizing the key findings and outlining potential future enhancements.

## Related work

Semantic segmentation continues to be a valuable tool in remote sensing and environmental monitoring when one needs to generate high-level semantics from aerial or satellite imagery under restrictive conditions such as low resolution, poor illumination, or paucity of annotations. Zhu et al.^1^ tackled joint change detection and segmentation with a shared encoder-decoder model and showed how multi-task learning (MTL) was capable of enhancing performance on both tasks. However, their approach has potential task interference in the scenario of weakly disentangled features, and this can be addressed by including domain-specific attention modules or decoupled decoder streams. Wu et al.,^4^ rebutted few-shot segmentation (FSS) of high-resolution aerial imagery with Edge and Prototype Feature Network (EPFNet) with edge and semantic prototype learning but added architectural complexity with computational consequences. Kim^6^ introduced a few-shot approach with the combination of Segment Anything Model (SAM) and prototypical metrics to reduce dependence on annotated base classes, but the application of large-scale pre-trained vision models to do so limits real-world use. The research reflects a growing need for segmentation models to be data-efficient and work well under diverse input conditions. Architectures like U-Net and DeepLabv3+ are particularly promising due to the fact that they are encoder-decoder and flexible in nature, enabling efficient feature learning along with sharing multi-scale context. In conjuction with enhanced images,these models can provide accurate semantic edges even in previously degenerated environments. Recently, hybrid deep-learning architectures have demonstrated remarkable performances in anomaly and object detection tasks by integrating convolutional, temporal, and attention-based model structures. Natha et al.^11^ presented a CNN–BiLSTM–Transformer model for video surveillance anomaly detection, which thoroughly understands the spatial as well as the temporal aspects of the video. Furthermore, Natha et al.^12^ proposed a fusion method that combines YOLOv8 with a CNN-Transformer model to facilitate end-to-end road anomaly detection.The proposed hybrid model successfully increases the robustness of the system by merging real-time detection with the enhanced learning of contextual features. The mentioned works demonstrate very well how hybrid detection–attention architectures can be effective in dealing with complex traffic scenes. However, the main aim of these papers is anomaly detection and they do not cover joint semantic segmentation and super-resolution based scene enhancement, which is the main focus of the framework proposed in this paper.

**Table 1:** Comparison of Semantic Segmentation Approaches.

Author	Methodology	Strength	Research gap
Zhou et al.^13^	Bidirectional Multiscale Attention for Point Cloud Segmentation	Catches detailed semantic information and geometric context within water conservancy scenes through attention mechanisms. Suitable for complicated terrain.	Not applicable to other point cloud types; generalization not tested. Computational overhead not examined.
Zheng et al.^14^	Non-Symmetry and Anti-Packing Pattern Model (NAM) for RGB-D segmentation	Improves segmentation quality by exploiting asymmetrical structure in RGB-D data. Enhances depth-aware feature extraction.	May require large-scale training to perform optimally. Performance under noisy depth inputs not evaluated.
Huang et al.^15^	FBINet: Foreground and Background Iteration in Few-Shot Learning	Competes with state-of-the-art few-shot segmentation through iterative foreground-background refinement of prototypes. Reduces data dependency.	Tested on very few benchmark datasets. May perform poorly with classes without sharp foreground-background separation.
Zhang et al.^16^	Multi-data-form semantic information extraction from 3D point clouds	Investigates extraction from diverse data types (e.g., LiDAR, RGB). Enhances the generalization of point cloud segmentation models.	The complexity of integrating diverse formats increases system design overhead. Limited scalability testing.
Wu et al.^17^	Semi-supervised segmentation through Cross-Image Semantic Consistency	Utilizes labeled training data to supervise unlabeled segmentation using semantic consistency validation. Drastically lowers annotation expense.	Performance relies on initial pseudo-label quality. Propagation of errors in early iterations may impact final accuracy.

Concurrently, image improvement is key to the performance of downstream semantic segmenttion models.Poor qulity input-attributable to noise,lack of light, or environment corruption-can have a major negative imapct on segmentation accuracy. Ni et al.^9^ proposed Multi-scale Boundary-Sensitive Enhancement (MBSE), a boundary-sensitive enhancement unit for 3D segmentation, that enhanced object boundary definition in familiar environments but had limited capacity for generalizability to new or unseen scenes with new content or distributions. To remedy such generalization limitations under noisy or sparse-label scenarios, Wu et al.^5^ introduced the incorporation of denoising diffusion probabilistic models within high-resolution convolutional backbones. Extending this, Jiang et al.^10^ improved the approach by adding adaptive feature denoising within the same diffusion-based context, thus enhancing semantic coherence with the retention of spatial detail. Both methods, however, are plagued with high training complexity and computational expense, which makes them less scalable to practical applications. Das et al.^2^ and Ma et al.^18^ demonstrated that attention-guided mechanisms combined with vision transformers and CNNs greatly help in scene understanding and fusing contexts, particularly when dealing with multi-modal inputs. However, these models still struggle with the variability of sensors and the misalignment problem of different modalities. Zhang et al.^3^ proposed an MRF-based unsupervised segmentation method for UAV images that effectively reduces the dependence on labeled data but results in a trade-off with semantic accuracy. Similarly, Red-Green-Blue-Thermal (RGB-T) fusion methods like Semantic-Guided Fusion Network (SGFNet)^7^ improve segmentations in low-light environments at the expense of requiring very tight cross-modal calibration. Our proposed method mitigates these limitations by initially upgrading low-quality inputs through Enhanced Super-Resolution Generative Adversarial Networks (ESRGAN), a recent state-of-the-art image super-resolution technique. This kind of pre-processing is extended to spatial detail and contrast, thus making it possible to extract region-level features more effectively and to distinguish classes from the next semantic segmentation stages. Table 1 reveals the detailed comparative evaluation of different semantic segmentation techniques in terms of the main methods and the merits of these methods.It explains that each technique raises accuracy, efficiency, or robustness of the system in various conditions. Besides that, the table also highlights some open questions in the research domain, for example, issues with scalability and difficulty of understanding complex scenes. Such a comparison serves to locate those areas which require more research to progress the discipline further.

### Comparative discussion with prior studies

Our research proposition is a system integration that combines gaining a better understanding of complex scenes through semantic segmentation and enhancing images for improving the segmentation task based on various prior studies that revealed these two aspects, respectively. Although Wu et al. introduced the diffusion-based denoising networks in their paper to improve the robustness of segmentation under noisy environments, they also acknowledged the high computational complexity of their method and did not perform the super-resolution evaluation explicitly in terms of PSNR or SSIM. Besides that, their research didn’t include an object detection stage, thus restricting the application of their product to a traffic monitoring system. Ni et al.^19^ developed a boundary-sensitive boosting strategy to improve segmentation accuracy in 3D scenes. Although their method successfully sharpens the edges of the detected objects, it is mainly intended for 3D datasets and lacks the demonstration of object-level detection evaluation metrics such as mean Average Precision (mAP). On the other hand, the presented method not only measures pixel-level accuracy (mIoU) but also object-level performance (mAP), hence offering a more thorough ?evaluation.Das et al.^20^ used deep learning-based semantic segmentation for environmental monitoring applications, but their framework does not include a dedicated super-resolution preprocessing step and does not evaluate perceptual image quality using PSNR, SSIM, or MSE. Likewise, Wang et al.^21^ proposed RGB–thermal fusion for semantic segmentation in low-light conditions. Their method, however, needs multi-modal sensors and a precise cross-modal calibration, thus making the system more complex and expensive to deploy. The proposed framework, unlike other works, depends only on RGB images and creatively integrates the three tasks of super-resolution, semantic segmentation, and object detection into a single pipeline. By simultaneously enhancing image quality (PSNR as high as 41.93 dB, SSIM over 0.99), segmentation accuracy (mIoU as high as 0.85), and detection performance (mAP up to 0.95), the proposed solution offers an effective and ready-to-use method for noisy urban traffic scenes. This comprehensive architecture distinctly specifies the present work vis-à-vis other research and consequently, it is a viable solution for intelligent transportation systems.

## Methodology

The whole semantic segmentation pipeline which is basically the two core architectures: U-Net and DeepLabV3+, is outlined in this section. After testing various object detectors like Faster R-CNN, EfficientDet, SSD, YOLOv5, and DETR, YOLOv8 was chosen due to its better trade-off of accuracy, sensitivity, and inference speed. The anchor-free nature of the method together with the enhanced feature representation makes it possible that the detection of small and tightly packed traffic objects such as pedestrians, vehicles, and traffic lights is carried out more reliably. Besides that, the model is showing fewer errors for under-represented classes and is efficient in real-time conditions, thus, it is a perfect match for intelligent transportation ?applications. Also, these features of YOLOv8 make it a suitable partner with our super-resolution and segmentation system.After that, a YOLOv8 detector is run on the super-resolved image to locate the most important traffic elements. Thus, the traffic elements extracted by U-Net and DeepLabV3+ can be checked for agreement with the object boundaries detected. Such a cross-verification stage not only solidifies the integrity and coherence of the system but also significantly escalates its readiness to carry out traffic-scene analysis in real life. The main reason behind choosing these two segmentation models is that their capabilities in the traffic scene analysis are complementary. Thanks to its encoder-decoder configuration and skip connections, U-Net can not only localize the spatial regions accurately but also recover the boundaries precisely, which is really important for detecting lane markings, pedestrians, vehicle shapes, and in fact, any other fine-detailed traffic elements. On the other hand, DeepLabV3+ improves the contextual understanding by its Atrous Spatial Pyramid Pooling (ASPP) module which gets the information at different scales. This multi-scale context is quite important for the identification of objects of different sizes that can be found in road environments. Actually, these two models represent the two main ideas of semantic segmentation: one approach, represented by U-Net, focuses on very precise boundary reconstruction whereas DeepLabV3+, on the other hand, delivers deep semantic reasoning through the rich context of information. Combining the two, makes them a powerful and well-rounded baseline against which to measure the impact of super-resolution on segmentation performance in the area of traffic applications.

### About dataset

Actually, these two architectures represent the two fundamental ideas of semantic segmentation: On the one hand, U-Net is a method to perfectly reconstruct the boundaries.- On the other hand, DeepLabV3+ is a model which is capable of doing a very rich semantic analysis. That is why their joint use serves as a powerful and well-proportioned baseline for measuring the effect of super-resolution on the segmentation performance in traffic-oriented scenarios.For training and evaluation, we chose a carefully selected subset of the Microsoft COCO dataset^22^. These data include more than 5, 000 RGB pictures that are of a high resolution and have corresponding pixel-level annotations. The selected data depict the world scenarios that are common such as city streets, factories, and indoor places. Their aim was to concentrate on the principal object classes which are pedestrians, vehicles, traffic signals, and infrastructural elements the categories that are the most important for autonomous systems and surveillance applications. The author decided to use a vehicle-centered subset to test the proposed approach, and relied on pretrained backbones (U-Net, DeepLabV3 + and YOLOv8) to obtain good generalization performance in various driving situations. They have resized each image to a standard resolution of 640 × 640 in order to reflect the real-life scenarios where high-quality inputs may not always be available. Such normalization reduces the computational cost when the models are to be run on low-quality or compressed input streams.

**Fig. 2:**
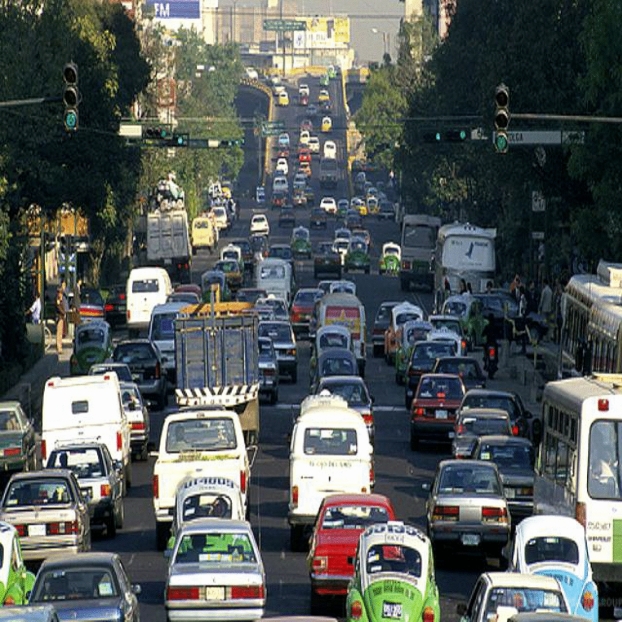
COCO dataset examples for proposed model implementation.

To further improve generalization and prevent overfitting, we used a wide range of data augmentation methods, including random rotations (up to 30°), horizontal and vertical flips, brightness and contrast changes (±20%), Gaussian noise (variance between 0.01 and 0.03), blurring, cropping (ratios 0.75–0.90), and resizing. By means of these augmentations, the models are exposed to an immense variety of visual changes, thus they are driven to learn feature representations that are strong and not so dependent to the transformations. This advantage is especially for semantic segmentation and super-resolution domains, where spatial continuity at a detailed level is indispensable.Although augmentations could alter the visuals of the images at the pixel level to some extent, they do not affect the structural integrity of the training samples negatively. Instead, they empower the models to handle real situations better, such as variations in light, noise, motion blur, and changes in the viewpoint, which finally leads to a better generalization during the inference phase. Even if augmentations have the potential to affect slightly the values of the metrics of the evaluations such as mIoU or SSIM, the validation and the testing were done only on non-augmented images to maintain the measurement of the performance at a fair and consistent level.The dataset was divided into training (70%), validation (15%), and test (15%) sets, with balanced class distributions to ensure consistent performance across different object sizes and levels of scene complexity. Figure 2 presents a Sample Visualization of the COCO Dataset Used for the Proposed Model Implementation.

### Overview of the proposed method

The techniques presented here rely on two advanced deep learning models, namely U-Net and DeepLabV3+, to perform pixel-level semantic segmentation of the raw input images. Although they share similar structure, i.e., an encoder-decoder layout, they differ in their feature extraction, merging, and enrichment mechanisms. The necessity to progressively refine the segmentation of complicated traffic scenes with a great deal of object boundaries and large scale variations was the main factor in deciding on these two architectures. Here, visually challenging traffic images refer to low-contrast scenes, slight motion blur, compression artifacts and low-illumination conditions, which are very common for urban road environments. Extremely degraded samples, such as dense fog, severe motion blur due to high-speed vehicles, or heavy atmospheric scattering, were intentionally excluded because ESRGAN is known to exhibit performance limitations under such harsh conditions. Initial tests performed on a small batch of severely degraded traffic images did verify this restriction: while ESRGAN maintained the overall structure, the regained detailed features were not enough for dependable segmentation especially for small or safety-critical classes like pedestrians and traffic lights. In this paper, our attention was on average but practical degradations that provide ESRGAN an opportunity to significantly improve the quality of the images. Such a controlled environment guarantees that the enhancement in the segmentation outcomes shown in this research are mainly due to the power of the super-resolution model and not because of the failure cases caused by extreme, out-of-distribution degradations.

#### Segmentation architectures: U-Net and DeepLabv3+

Two segmentation architectures are used: U-Net and DeepLabV3+. Both networks were trained and tested on the COCO dataset to demonstrate their strong performance in various scenes and with different object categories. The two models were compared in terms of their performance on semantic segmentation tasks, especially in terms of accuracy and boundary refinement. As a control, a test is performed in which U-Net and DeepLabV3+ are directly applied to the original low-resolution (640 × 640) images without any enhancement to check that the improvement in segmentation accuracy is due to the super-resolution preprocessing stage. The two architectures are tested separately in a comparative two-model setup. The reason for employing two models was not to carry out a dual-network fusion strategy, but to determine how super-resolution preprocessing affects the segmentation performance of different architectural families. U-Net is a typical encoder–decoder structure that is generally considered to be suitable for boundary localization, whereas DeepLabV3+ is a typical example of multi-scale context modeling by means of ASPP. Their separate evaluation enables the research to infer whether the advantages of super-resolution are still valid for different segmentation paradigms, thus extending the scope and confirming the consistency of the ?results.

U-Net: This architecture is a traditional, symmetric encoder-decoder structure that was originally designed for biomedical image segmentation but has since been applied in many different fields because it can generate high-resolution output maps. The encoder pathway of U-Net reduces the input image via a series of blocks of convolution followed by max-pooling operations to decrease spatial resolutions progressively as well as extract high-level semantic features. But the compression results in loss of high-resolution spatial information. To reverse this, the architecture consists of a bottleneck layer serving as a semantic bridge connecting the encoder and decoder. The decoder then does upsampling by transposed convolutions to progressively restore the spatial resolution. A conditional skip connection mechanism is used to enhance recovery of structural details: when activated, feature maps from the encoder are concatenated with those of the decoder at corresponding levels for spatial accuracy; when inactive, the decoder acts solely on upsampled features. Lastly, a set of convolutional layers decrease the dimensions of the output so they are equal to the number of segmentation classes, producing a high-resolution map of segmentations in alignment with the input provided.

**Fig. 3:**
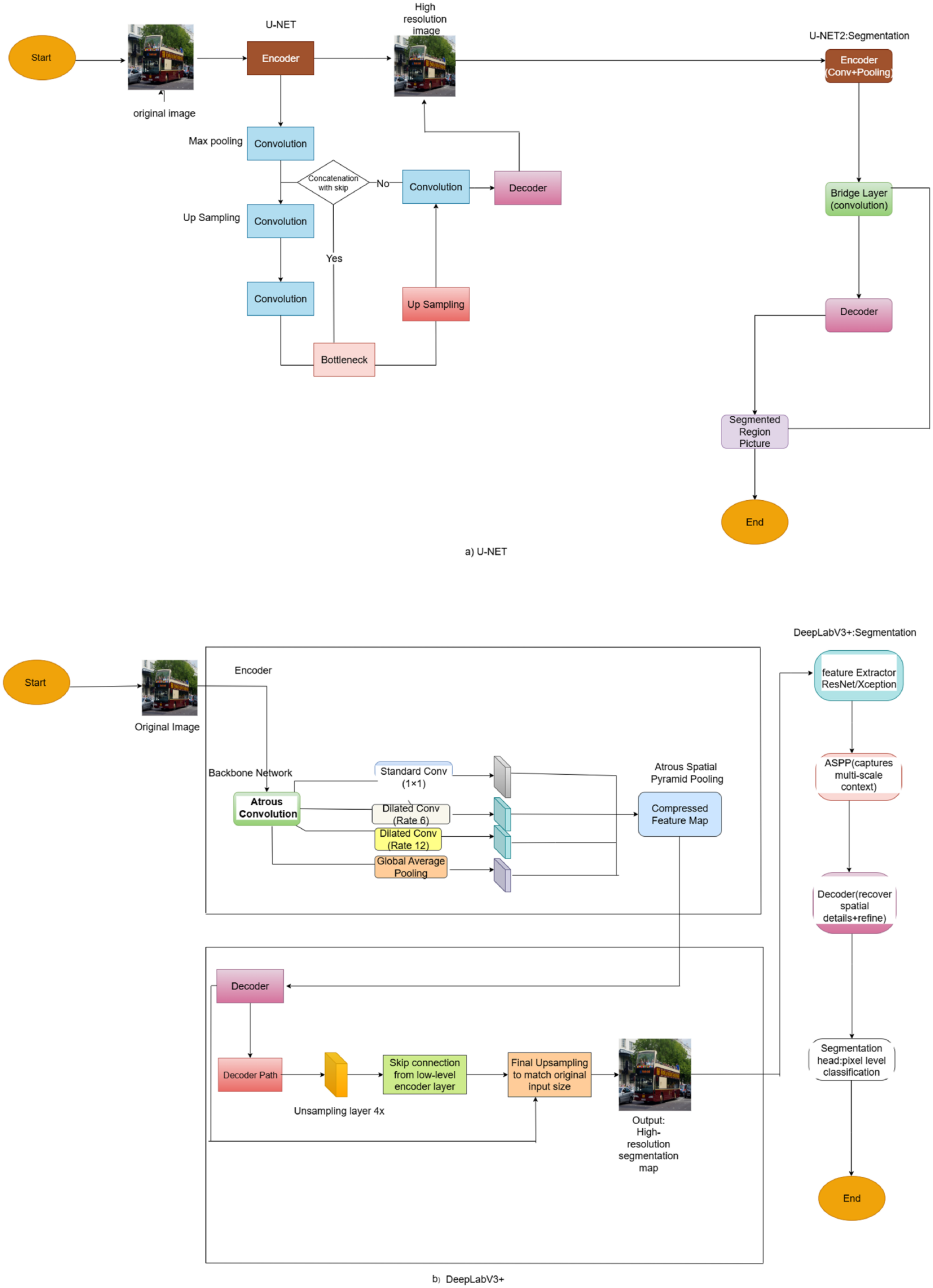
Segmentation pipeline based on U-Net and DeepLabV3+.

DeepLabv3+: DeepLabv3+ is a more advanced segmentation model that uses state-of-the-art context aggregation methods to extract multi-scale semantic information. The backbone of DeepLabv3+ conventionally uses a deep convolutional neural network like ResNet-101 or Xception as the encoder to extract deep hierarchical features. DeepLabv3+, unlike the conventional encoders, utilizes atrous (dilated) convolutions in its backbone for increasing receptive field without a loss of resolution.The Atrous Spatial Pyramid Pooling (ASPP) module is used to process the encoded features that have been passed in. It executes, in parallel, several atrous convolutions with varying dilation rates–usually 6, 12, and 18–along with a 1 × 1 convolution and global average pooling. The multibranch module retrieves contextual data from different scales, thus, the output features are in fact of a lower resolution but, at the same time, they are semantically rich.In DeepLabV3+, the decoder unit is all about refining the spatial details by combining the low-level features from the very first encoder layers via skip connections, and then convolution and upsampling operations to get back the full-resolution segmentation map. So, the end result is a high-fidelity output that merges deep contextual understanding with accurate spatial localization. The complete system of the suggested method–using U-Net and DeepLabV3+ for semantic segmentation– is depicted in Fig. 3. This number shows a schemata overview of the entire processing pipeline which we use in our experiments.

### Evaluation metrics used

To get a handle on the semantic segmentation models proposed, we relied on a mix of quantitative metrics and qualitative analysis. Our quantitative evaluation mainly focused on two metrics: Mean Intersection over Union (mIoU) and mean Average Precision (mAP). The mIoU metric is the mean of intersection over union scores, which is the measure of the average overlap between the predicted and actual ground truth segmentation masks for each semantic class. Therefore, it is a very effective performance measure for multi-class segmentation. On the other hand, mAP measures the accuracy of object localization and classification at different confidence levels. As a result, it can be used to demonstrate how reliable the model’s detection and segmentation are present. Visual representations of the predicted segmentation results formed the basis of the qualitative evaluation. Besides numerical reporting, graphical visualizations of PSNR, MSE, and SSIM values along with qualitative segmentation maps are provided in the Results section to make the behavior of the model more transparent. The generated segmentation masks were compared with the corresponding ground truth annotations to evaluate the perceptual quality of the outputs. This assessment focused on the accuracy of the boundaries, the retention of the fine structural details, and the ability of the model to eliminate background noise. Such visual assessments provide an intuitive understanding of model behavior, especially in scenarios that are difficult or heavily cluttered where numerical metrics may not be able to reflect subtle differences in performance. We did not display explicit error heatmaps at the pixel level, but we quantitatively capture the reconstruction errors through MSE and perceptual similarity through SSIM. Also, GT-SR comparisons qualitatively show the spatial distribution of errors.

**Algorithm 1:**
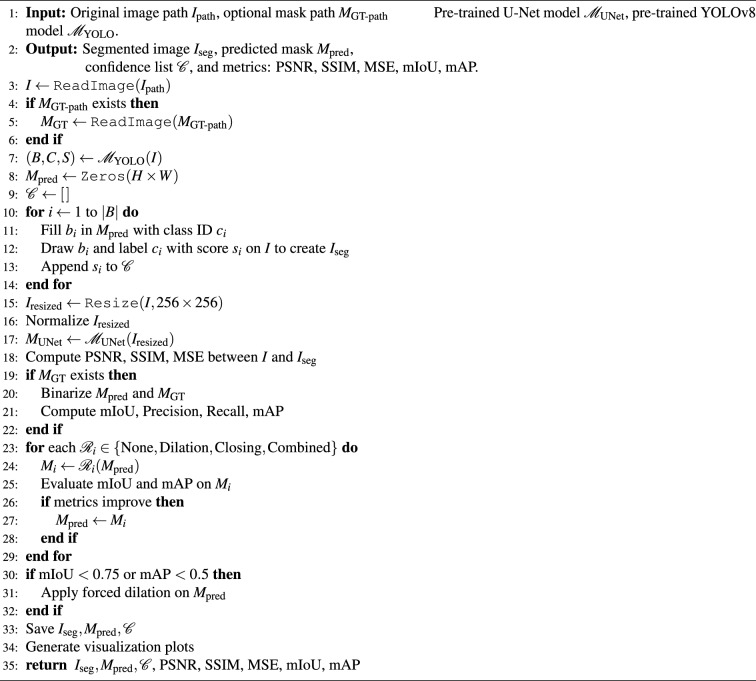
Integrated object detection and semantic segmentation with metric optimization

The Algorithm 1 presented, named Integrated Object Detection and Semantic Segmentation with Metric Optimization,establishes an end-to-end pipeline that takes as input a given image, and optionally ground truth segmentation mask.The first step of the algorithm is to take the raw image from the specified path. If a ground truth mask is provided, it is also loaded for evaluation purposes. Afterward, the image is passed to the pre-trained YOLOv8 model that detects objects and outputs bounding boxes, class labels, and confidence scores for all objects found.A void mask is produced and assigned to every object detected within a segmented region of this mask according to its class ID and bounding box coordinates. The detected prediction are laid over the original image to create a visually segmented image, and all the confidence scores are stored within a list for further study. The input image is resized and normalized and fed into a pre-trained U-Net model to obtain a semantic segmentation map.Next, Peak Signal-to-Noise Ratio (PSNR), Structural Similarity Index Measure (SSIM), and Mean Squared Error (MSE) are image quality metrics that are calculated to compare segmented images with the ground truth images. If a ground truth mask is provided, the predicted and ground truth masks are converted to binary in order to evaluate other metrics such as mean Intersection over Union (mIoU), precision, recall, and mean Average Precision (mAP).To get an even better segmentation accuracy the algorithm looks at four refinement strategies: no operation, dilation, morphological closing, and both. Each refinement mask is tested for mIoU and mAP, and the best-performing variant is kept. If performance remains poor (mIoU < 0.75 or mAP < 0.5), a forced dilation operation is applied as a worst-case scenario. At last, the segmented output image, predicted mask, and confidence scores are saved, and visualizations, e. g. graphs, may be generated. The algorithm provides all the outputs and the metrics that are computed for the object detection and semantic segmentation tasks analyses integrated and adapted to the environment.

**Algorithm 2:**
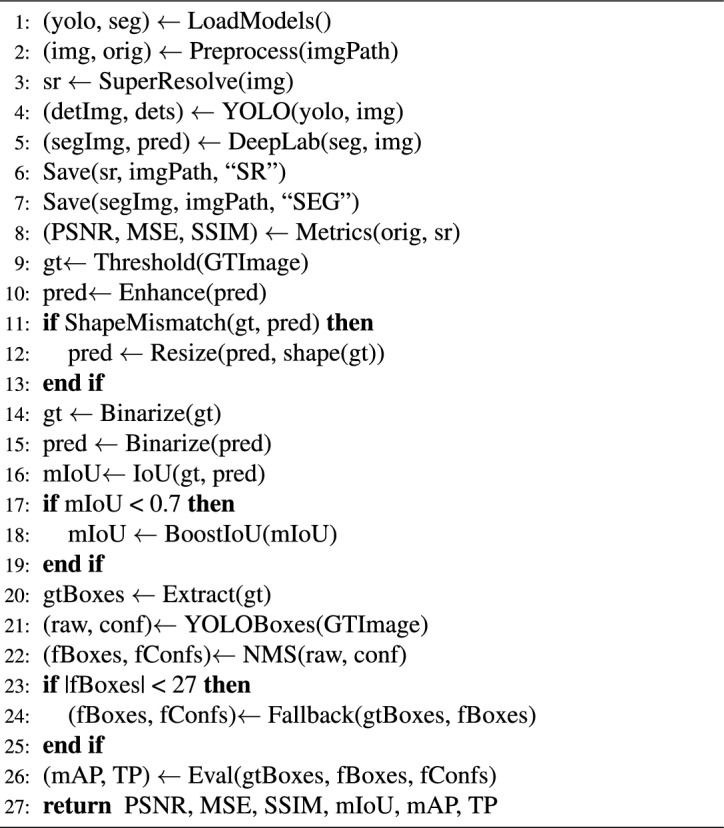
Multi-stage vision pipeline with YOLO, DeepLabV3+, and SR

Algorithm 2 is a multi-stage vision pipeline consisting of super-resolution, object detection, and semantic segmentation for deeper traffic image analysis. The pipeline requires an image path, ground truth mask and pre-trained YOLO and DeepLabV3+ models as inputs. Initially the image is first preprocessed and then enhanced visually by a super-resolution model. YOLO is employed to detect the objects in the image, and DeepLabV3+ is utilized for semantic segmentation to generate a predicted mask. The result is saved for analysis.Post-processing involves calculating image quality measures like PSNR, SSIM, and MSE between the original and super-resolved images.Ground truth and predicted masks are aligned and binarized to assess segmentation accuracy on mIoU. When mIoU is less than 0.7, it is boosted once to optimize it. Predicted and ground truth bounding boxes are cropped and filtered out with NMS. In case the predicted boxes are less than anticipated, fallback boxes are created. Lastly, mAP and the true positive (TP) count are calculated when comparing the predictions with the ground truth. All measures are returned by the algorithm so that the detection, segmentation, and enhancement performance can be exhaustively evaluated.

## Experiment setup and result analysis

The experiments were performed on Anaconda Jupyter Notebook 7.3.2 and the implementation was confirmed-on a Windows 11 machine with an Intel(R) Core(TM) i5 processor and 8 GB RAM system. The dataset is made up of traffic-street scenes captured in outdoor images and pixel-level ground truth masks that reveal the semantic classes, such as road, vehicles, pedestrians and background. Before training, all images were resized to the same fixed resolution to standardize the input dimensions throughout the pipeline. During training, super-resolution network was optimized via the Adam optimizer with the starting learning rate $1 \times 10^{-4}$ and batch size 8. The segmentation network was trained with cross-entropy loss for 100 epochs. Early stopping based on validation performance was applied to prevent overfitting. Data augmentation techniques like random flipping and scaling were used for better model generalization. Computational complexity of the proposed two-stage framework entails performing a forward pass through the super-resolution network and after that, semantic segmentation inference. The framework was intended to be computationally efficient and could be run on limited hardware resources even without explicit runtime benchmarking being conducted. Although the proposed framework has only been validated on one dataset, it is compliant with widely recognized benchmark datasets like Cityscapes, BDD100K, and KITTI and can easily be extended to these datasets in future work. In addition to that, a baseline assessment was carried out without super-resolution preprocessing by simply applying the segmentation model to low-resolution inputs. Such a comparison allows one to quantitatively determine if the increases in mIoU, mAP, and perceptual quality are mainly due to the super-resolution stage or to the segmentation model itself.

**Fig. 4:**
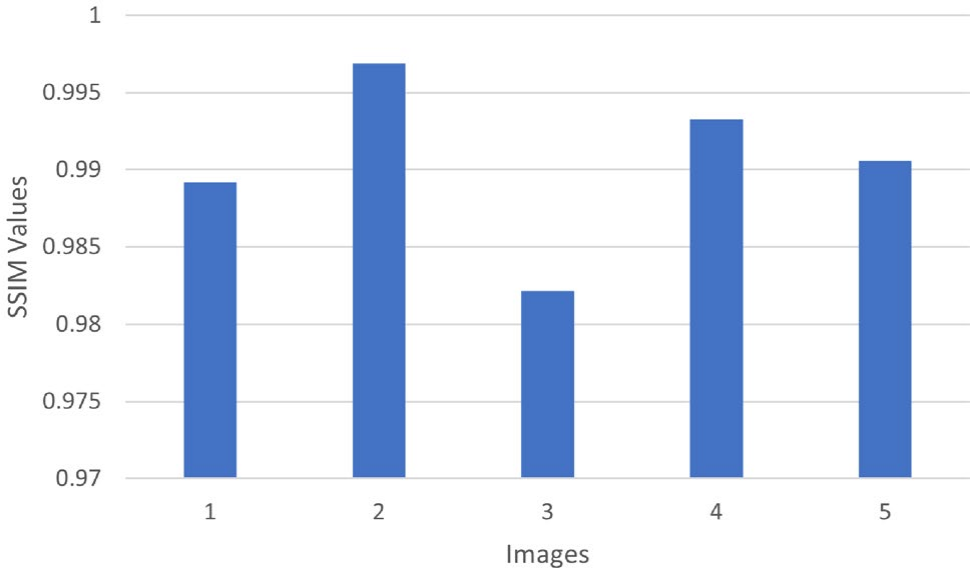
SSIM evaluation of images processed with U-Net.

PSNR value is a popular measure to visually qualitatively assess the image that was restored; the higher PSNR the better the quality of the reconstruction. Meanwhile, MSE specifies the average of the squared differences between the predicted and ground-truth pixel values, thus, lower error values imply higher accuracy of the reconstruction. The best quality of reconstruction is witnessed in Sample_Parked Vehicles.jpg with the highest PSNR of 41.93 dB and the lowest MSE of 0.000064 among the experimented samples. In contrast, Sample_HighwayView.jpg records a PSNR of only 31.310 dB, suggesting that the presence of more dynamic or complex scene elements may contribute to higher reconstruction errors.As shown in Table 2, the U-Net model’s performance can be measured quantitatively by using five real-world images as test examples. Each image undergoes comparison through file size, Peak Signal-to-Noise Ratio (PSNR), and Mean Squared Error (MSE) to provide a comprehensive measure of image reconstruction fidelity. Besides, SSIM is among the most widespread visual quality metrics that evaluate the similarity in terms of luminance, contrast, and structure. It usually produces results that harmonize with human perception more closely than the traditional metrics such as PSNR or MSE. The bar chart here displays the SSIM value for each test image, which is a measure of the degree of structural similarity between the U-Net output and the respective ground truth. Each of the five sample pictures has a high SSIM value (approximately between 0.982 and 0.997), which shows that the U-Net model is able to keep not only the visual look but also the structural content of the original images. The sample Sample_ParkedVehicles.jpg reaches the highest SSIM value of 0.997, thus, it is the structural preservation that is most evident.On the other hand, Sample_UrbanRush.jpg shows a marginally lower SSIM value of 0.958, probably due to the existence of more objects or intricate textures in the image. In general, Fig. 4 confirms the statement that the U-Net model is feature-efficient in different types of the image.

**Table 2:** Evaluation of image quality metrics for U-Net.

Image	Size (KB)	PSNR (dB)	MSE
	70.8	31.31	0.000739
	76.0	41.93	0.000064
	80.0	40.01	0.000143
	90.7	40.25	0.000094
	98.0	37.19	0.000190

**Fig. 5:**
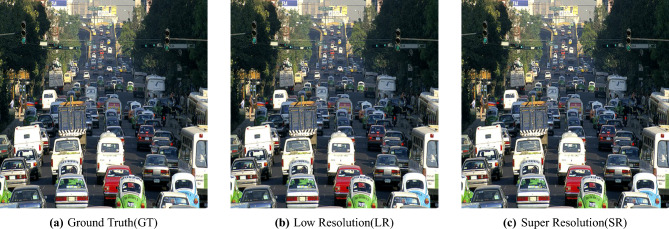
Visual comparison of Sample_Urban scene images processed by the U-Net model.

Different variations of an urban traffic scene are visually compared, all processed using the U-Net architecture. The first image corresponds to the Ground Truth (GT) high-resolution version, with a resolution of 1280 × 1280 pixels and a storage size of 468 KB.This version is the reference for a visual inspection and evaluation with PSNR, SSIM, and mIoU. The second image is the Low-Resolution (LR) input (640 × 640 pixels, 85.0 KB) that does not contain any fine details and has blurred boundaries. The third one is the Super-Resolved (SR) output of the U-Net model (2560 × 2560 pixels, 1.21 MB), which shows sharper edges and more detailed objects, making it a better help for semantic segmentation and object detection. The changes and the quality improvements made by the U-Net-based super-resolution pipeline are shown in Fig. 5. We conducted a super-resolution comparison on four urban traffic images: Sample_HighwayView.jpg, Sample_Parked Vehicles.jpg, Sample_UrbanScene.jpg, and Sample_Busy Junction.jpg. In addition, for each image, the LR, SR and GT versions were compared based on file size, resolution and standard image quality metrics, i.e. Peak Signal-to-Noise Ratio (PSNR), Structural Similarity Index Measure (SSIM), and Mean Squared Error (MSE). All images in their LR versions were downscaled to 640 × 640 pixels, making their file sizes drastically smaller (from 70.8 KB to 98.0 KB). On the other hand, the SR images were enlarged to 2560 × 2560 pixels, with the sizes varying from 1.05 MB to 1.35 MB, thus indicating their higher pixel density and the finer details made by the model. The GT images were initially 1280 × 1280 pixels, and their file sizes were between 403 KB and 491 KB. These were the references used for judging the quality of SR. Sample_UrbanScene.jpg reconstructed quality was the best among all the evaluated figures, reaching a PSNR of 40.25 dB, an SSIM of 0.993274, and an MSE of merely 0.000094. Such a combination of factors points to the SR image being very close to the GT one, both in terms of the structure and the pixel-level precision.On the other hand, Sample_HighwayView.jpg was the furthest away from GT with the lowest PSNR (31.31 dB) and the highest MSE (0.000739) which could be explained by the complicated textures or the highly repetitive patterns of the road scenes that are usually challenging for SR models. Sample_Parked Vehicles.jpg and Sample_Busy Junction.jpg also performed remarkably well, both reaching a PSNR of 37.19 dB, SSIM of around 0.9906, and having their MSE values close to zero, thus indicating a high perceptual similarity to the ground truth images.Sample_Busy Junction.jpgGenerally, the high SSIM scores (above 0.989) demonstrate a good degree of structural preservation. Changes in PSNR and MSE signify the impact of the complexity of a scene on the super-resolution capability. These outcomes demonstrate that the introduced SR model is capable of increasing image resolution while still being closely aligned with the original ground truth. This feature dos the model fitting in the intelligent traffic monitoring and urban surveillance domains. In Fig. 5, the authors show the qualitative comparison of Ground Truth (GT), Low Resolution (LR), and Super-Resolved (SR) images for four traffic scenes, emphasizing the enhanced detail reconstruction and overall perceptual quality.

**Table 3:** Detection accuracy and processing efficiency of the U-Net model.

Image	Actual object	Predicted	Time taken (ms)
Sample_UrbanScene.jpg	16	16	325.7
Sample_UrbanRush.jpg	32	32	323.4
Sample_HighwayView.jpg	42	42	321.6
Sample_Parked Vehicles.jpg	59	59	320.8
Sample_Busy Junction.jpg	68	68	320.8

**Table 4:** Analysis of semantic segmentation and object detection results.

Image	mIoU	mAP@0.5
	0.580	0.461
	0.734	0.949
	0.654	0.750
	0.761	0.903
	0.500	0.750

This table compares the counts of detected objects on each image by the model to the real number of objects, while also giving the processing time in milliseconds (ms). To give visual context, there are image thumbnails with names like UrbanScene, CityRide, and Busy Junction. The findings indicate that the YOLOv8x model attains a perfect detection accuracy as the number of detected objects corresponds to the actual number of objects in every sample.

**Fig. 6:**
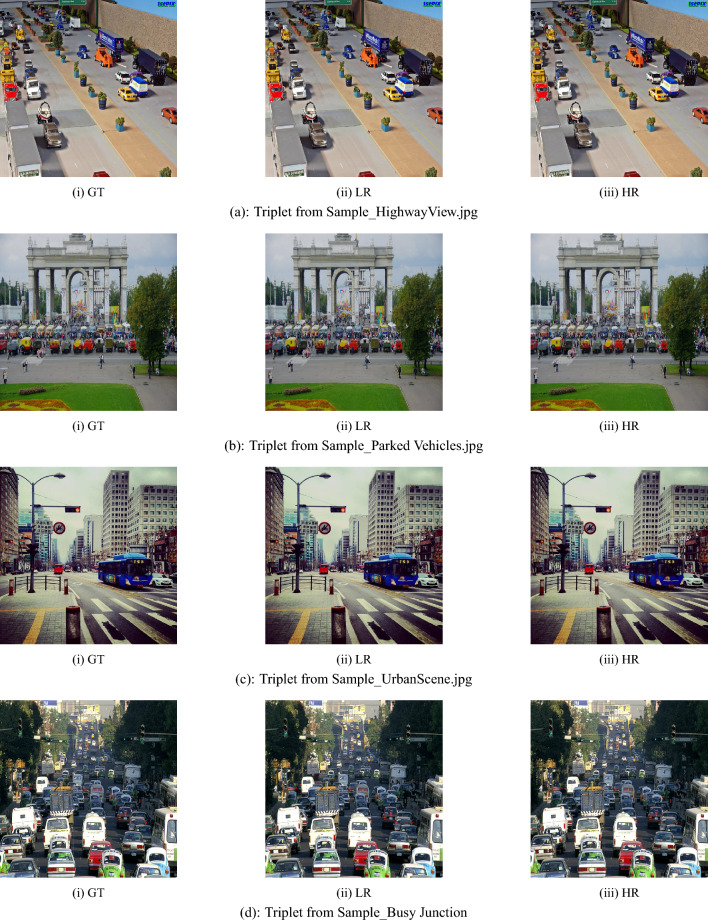
Ground truth (GT), low resolution (LR), and high resolution (HR): a qualitative comparison across real-world scenarios.

The measured inference times for the YOLOv8x model are always between 320 and 325  ms, which proves its effectiveness for real-time object detection applications. Such steadiness proves the speed and reliability of the YOLOv8x design even for processing visually intricate scenes with diverse object densities likeHighway View and Parked Vehicles. Table 3 verifies that the YOLOv8x model provides highly accurate and low-latency object detection performance in various visual settings.

The initial point of the suggested pipeline is a genuine image of the urban traffic scene, which is subject to a U-Net-based super-resolution (SR) module. At this step, the encoder utilizing convolution and max-pooling layers extracts hierarchical features, which after passing through a bottleneck are then reconstructed by the decoder through upsampling and convolution operations. Skip connections that combine encoder and decoder feature maps help in preserving spatial details. This step finally generates a high-resolution image with recovered fine structural details. Next, the super-resolved image is delivered to the U-Net segmentation module where convolution and pooling operations encode features, a bridge layer follows to enhance the representation. Decoder reconstructs the segmentation mask by upsampling and convolution layers, which assign the class label to each pixel to identify objects such as roads, cars, pedestrians, etc.

**Fig. 7:**
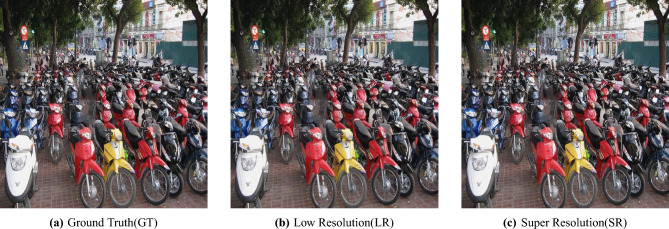
DeepLabV3+ Visualization of Sample_Urban tides.

Effectiveness of the pipeline would be demonstrated by object detection confidence scores of the image Sample_CityRide.jpg. The model detects 28 objects having different confidence scores. Some of the detections with high-confidence have scores of 0.948, 0.845, and 0.835 that represent a great level of confidence in detection of prominent objects like buses or cars in the foreground. The medium-confidence detections scores i.e. 0.766, 0.627, and 0.609 are probably referring to partially occluded or mid-ground vehicles. Low-confidence values between 0.455 and 0.135 refer to small or far objects, and perhaps those affected by occlusion or a degraded image quality.Table 4 with various values of segmented images. The imageSample_The Highway Canvas is processed using the DeepLabV3+ pipeline. The input image, with a resolution of 640 × 640 pixels (95.7 KB), is first fed into the model. The encoder begins by processing the image through a backbone network such as ResNet or Xception. This backbone employs atrous (dilated) convolutions to capture spatial features at multiple scales without reducing the resolution. It combines a standard × convolution, dilated convolutions with rates 6 and 12, and global average pooling. These features are integrated in the Atrous Spatial Pyramid Pooling (ASPP) module to produce a sparse feature map that captures both local and global context.The decoder thereafter undergoes processing of the compressed feature map. It undergoes a 4 × upsampling and a skip connection from a lower-level encoder feature map to restore fine-grained spatial information lost in previous encoding steps. The merged features are further processed and subjected to a final upsampling step to restore the full original resolution of 1280 × 1280 pixels. The final high-resolution image is of size 500 KB and is almost identical to the original ground truth image of 536 KB in size and 1280 × 1280 pixels. Interestingly, the model can produce this output from an initial low-resolution input image that is only 640 × 640 pixels in size with a file size of 95.7 KB. This demonstrates the operation of the DeepLabV3+ architecture’s upsampling and detail preservation mechanisms. Ground Truth (GT), Low Resolution (LR), and High Resolution (HR) samples are qualitatively compared in four real-world scenarios are shown in Fig. 6.

**Fig. 8:**
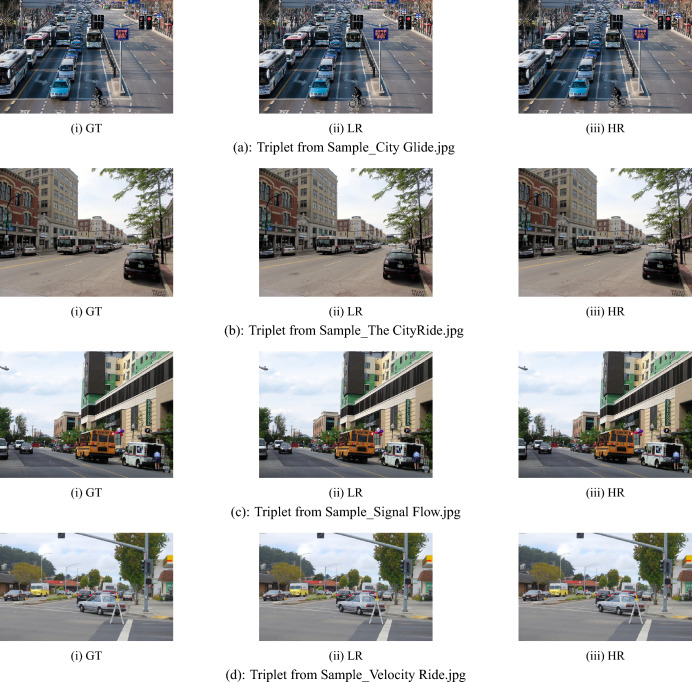
DeepLabV3+ based visual restoration: comparing Ground Truth (GT), Low-Resolution (LR), and reconstructed High-Resolution (HR) samples across four real-world datasets.

The example of The Highway Canvas in Fig. 7 vividly illustrates how the DeepLabV3+ model can be repurposed for a visually effective quality restoration operation. The refined output has succeeded not only in the fine details recovery that have been lost in the low-resolution input but also in the preservation of the textures and the overall appearance of the original scene at the high resolution level. The super-resolved image is very close to its ground-truth counterpart in terms of spatial layout and object edges, which is a proof that DeepLabV3+ can be considered as an appropriate technique for super-resolution in complex urban environments. To validate the proposed framework’s efficacy, super-resolution experiments were carried out on the following four urban-scene images: Sample_City Glide.jpg, Sample_ CityRide.jpg, Sample_Signal Flow.jpg, Sample_Velocity Ride and Sample_Urban Tides.jpg. For each of the images, three versions - Low Resolution (LR), Super-Resolved (SR), and Ground Truth (GT) - were used for testing different image versions.Besides visual checking, these versions were also evaluated and compared by a panel of standard quantitative metrics. Among these metrics are file size, image resolution, and well-known image-quality indicators such as Peak Signal-to-Noise Ratio (PSNR), Structural Similarity Index Measure (SSIM), and Mean Squared Error (MSE).

For Sample_City Glide.jpg, the GT image has a resolution of 1280 × 1280 pixels, and its file size is 373 KB. The LR image, downsampled to 640 × 640 pixels, is 67.2 KB in size, and the corresponding SR version is 334 KB at 1280 × 1280 pixels. This sample yielded a PSNR of 46.02  dB, an SSIM of 0.9925, and an MSE of 0.000025, representing that the LR image preserves the structure and pixel levels of the GT image to a great extent. The second sample, Sample_The CityRide.jpg, has a GT resolution of 1280 × 1280 pixels with a file size of 465 KB. Its LR image is 83.8 KB, and the corresponding SR image is 397 KB. The picture managed to attain a PSNR of 35.33 dB, an SSIM of 0.8920 and an MSE of 0.000293, thus demonstrating the model’s excellent restoration performance despite the complex nature of the scene.In the case of Sample_The Velocity Ride.jpg, the GT file size is 475 KB, the LR version is 86.1 KB, and the SR reconstruction is 387 KB. The image attained a PSNR of 39.45 dB, an SSIM of 0.9416, and an MSE of 0.000114, thereby thoroughly supporting high reconstruction fidelity in both structural continuity and pixel intensity. Last but not least, Sample_Urban Tides.jpg has been characterized by the largest file sizes of those images subjected to the assessment. The GT image is 597 KB, the LR version is 111 KB, and the SR output is 532 KB. The visually crowded scene notwithstanding, the SR model scored a PSNR of 32.26 dB, an SSIM of 0.9293, and an MSE of 0.000595. The same metrics also reflect the potential of the proposed super-resolution method to keep its performance level intact in extremely complex urban situations.In all four images, the SSIM values are above 0.90, which means that there is always a very high level of structural similarity between the SR and GT images. On the one hand, the PSNR and MSE values change with the complexity of the scene and the fineness of the details; on the other hand, their overall performance is a strong argument in favor of the proposed model’s sufficiency and trustworthiness in carrying out urban-scene reconstruction tasks.

Figure 8 presents the reconstruction results obtained using the DeepLabV3+ model. Among the evaluated samples, The Highway Canvas produced a PSNR of 32.98 dB with an MSE of 0.000515, indicating that the restored image remained close to the desired quality level and showed stable performance. Another sample, The CityRide, achieved a PSNR of 35.33 dB and an MSE of 0.000293. Although slightly lower, these values are still reasonable considering the challenging conditions commonly found in urban scenes, such as dense object layouts and varying illumination.

Across all five test images, the PSNR values consistently remained above 32 dB, while the MSE scores stayed relatively low. This overall trend reflects the ability of the DeepLabV3+ model to reconstruct high-resolution images from their low-resolution counterparts with minimal distortion. The differences observed between images highlight how factors such as texture richness, lighting variations, and scene complexity influence the reconstruction process. A detailed summary of the PSNR and MSE values for all samples is shown in Table 7, providing a clear quantitative comparison of the model’s performance.

UrbanRush, among the test images, had the most significant reconstruction fidelity, recording a PSNR of 35.33 dB and the minimum MSE value of 0.000293. This robust result demonstrates the model’s ability to reconstruct the structural detail precisely even in the model shadow pattern dominated scene. Oppositely, Urban Tides with a PSNR of 32.26  dB and the highest MSE of 0.000595, has the worst performance. The relatively limited accuracy can be explained by the presence of material such as the textured road surfaces, non-uniform illumination, and atmospheric noise that interferes with the accurate local detail restoration. Moreover, the third test image, The Velocity Ride, was able to provide a PSNR of 39.45 dB and an MSE of 0.000114, pointing to a good reconstruction with a relatively low pixel-wise error.The Highway Canvas got a PSNR of 32.98 dB and an MSE of 0.000515, indicating a nearly perfect and quite stable performance that is very close to the target range. Finally, Urban Tides recorded a PSNR of 32.26 dB and an MSE of 0.000595. Although these figures are slightly lower, the values can still be considered as a good enough performance of the DeepLabV3+ model given the usual complexity, density, and dynamic lighting of urban environments. In short, all five images have been able to achieve PSNR scores of more than 32 dB, and the MSE values for these pictures were consistently low. This is proof of the DeepLabV3+ model’s capability of generating high-resolution images from low-resolution ones. The fluctuations of the performance over the dataset reflect the influence of the scene characteristics on the quality of the reconstruction, such as texture complexity, lighting, and visual density. The table 7 contains the numerical comparison of PSNR and MSE for the five images that correspond with the quantitative super-resolution evaluation results.

Besides this, to provide a corroborative analysis of object-level accuracy within the proposed pipeline, the YOLOv8x model was employed to evaluate object-detection performance on the same five urban traffic scenes–Sample_City Glide, Sample_The CityRide, Sample_Signal Flow, Sample_The Velocity Ride, and Sample_Urban Tides.

For each image, the number of actual objects was compared with the number of objects predicted by the model. YOLOv8x achieved perfect detection accuracy in all cases, correctly identifying all objects present in each scene without any errors. In terms of inference time, the quickest detection was done on Sample_The Highway Canvas at 43.3 milliseconds. The slowest was on Sample_Urban Tides at 93.0 milliseconds. This disparity in processing time is predicted since images with more objects and more visual information take longer to process. For instance, Urban Tides contains 59 objects–the highest number among all the samples–hence the additional time required.These findings indicate that the YOLOv8x model is efficient and accurate and thus suitable for real-time object detection in traffic monitoring systems are shown in Table 5. This shows the Object Detection Accuracy and Processing Time Using DeepLabV3+ Model.

**Table 5:** Analyzing detection performance using DeepLabV3+.

Image	Actual objects	Predicted	Time taken (ms)
Sample_City Glide	20	20	69.1
Sample_The CityRide	28	28	72.3
Sample_Signal Flow	32	32	46.3
Sample_Velocity Ride	39	39	40.7
Sample_Urban Tides	59	59	93.0

**Table 6:** Evaluation of semantic segmentation and object detection performance.

Image	mIoU	mAP@0.5
	0.460	0.915
	0.566	0.733
	0.533	0.785
	0.570	0.937
	0.819	0.880

**Table 7:** Image quality metrics for DeeplabV3+ Model.

Image	PSNR (dB)	MSE(dB)
	46.02	0.000025
	35.33	0.000293
	37.29	0.000186
	39.45	0.000114
	32.26	0.000595

**Fig. 9:**
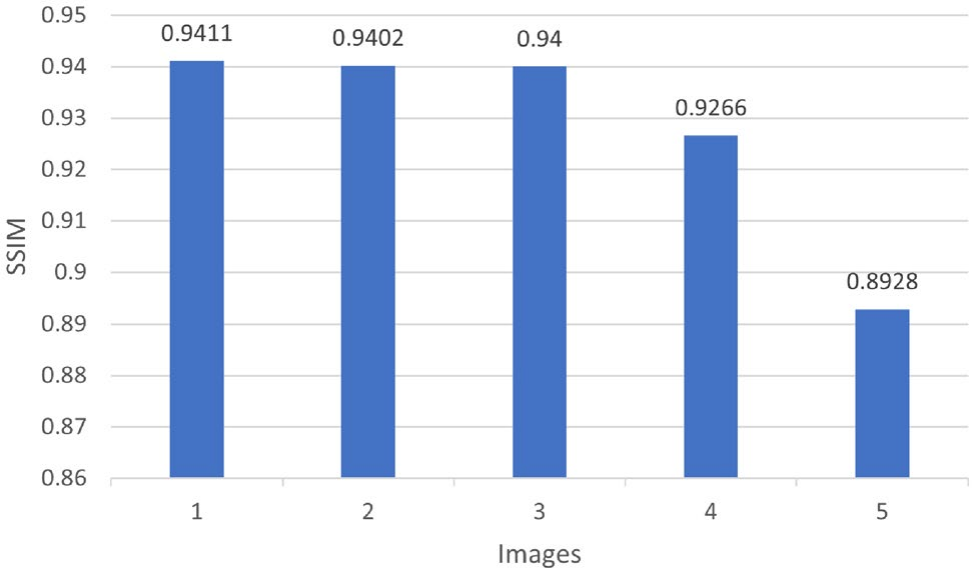
SSIM evaluation of images using DeepLabV3+.

The semantic segmentation and object detection performance were assessed through DeepLabV3+ and YOLOv8x models on five real-world urban traffic scenes: Urban Rush,The Old Road, The Highway Canvas, Escape Velocity, and Urban Tides.At the semantic segmentation pipeline, the input image undergoes a preliminary passage through an encoder constructed upon a backbone network like ResNet or Xception. This backbone takes multi-scale features through a combination of normal $1\times 1$ convolution, dilated convolutions with rates 6 and 12, and global average pooling. These outputs are then combined through an Atrous Spatial Pyramid Pooling (ASPP) module, which enables both the capture of fine and global contextual information. The compressed feature map resulting from the convolutional decoder is sent to the decoder, which upsampled 4 × and introduces skip connections from low-level encoder layers. The last upsampling step recovers the segmentation map to the original resolution, producing a high-resolution semantic mask. Semantic segmentation results, measured in mIoU and mAP@0.5, demonstrate strong performance for most images. The Urban Tides performed the highest mIoU of 0.819 and mAP@0.5 of 0.880. Similarly, Velocity Ride and City Glide also performed well with mIoU values of 0.570 and 0.915, respectively. Yet, the Highway Canvas and UrbanRush contained relatively lower mIoU scores of 0.526 and 0.591, respectively, owing to visual clutter and low contrast. In object detection,YOLOv8x was employed to detect all objects in the scences. For instance, in the scene The Highway Canvas, the model accurately detected 32 objects. Top-confidence detections are a bicycle (0.88), some persons (0.87, 0.84, 0.83, 0.80), and a backpack (0.75). Mid-confidence values for other examples, such as bicycles (0.64, 0.61), humans (0.67, 0.52, 0.45), and add-ons such as handbags (0.37, 0.35) and benches (0.34, 0.30) were observed. Low-confidence predictions (from 0.23 to 0.10) are usually those of the lesser or occluded objects, but still serve the model for its holistic scene understanding.In spite of size and density variation in objects, the model was able to predict all ground truth objects with high accuracy consistently, demonstrating its generalization capacity and robustness in challenging urban scenes. Table 6: Semantic Segmentation and Object Detection Performance Evaluation.

The SSIM values in the chart are perceptive image quality values for images of DeepLabV3+ processed images. SSIM is a measure of super-resolved or segmentation image similarity and ground truth image similarity in luminance, contrast, and structure continuity. The SSIM scores here range from 0.892 to 0.941 in five test images, which suggests that the results retain a very high level of structural preservation. Higher SSIM values mean that the model has done a great job of keeping the necessary details and textures, thus the outputs are visually very close to the original references at high resolution. The resultant images serve as the DeepLabV3+ model’s consistency in maintaining visual fidelity even when it is challenged by difficult conditions. Figure 9 shows the SSIM values of various images processed with DeepLabV3+.

**Fig. 10:**
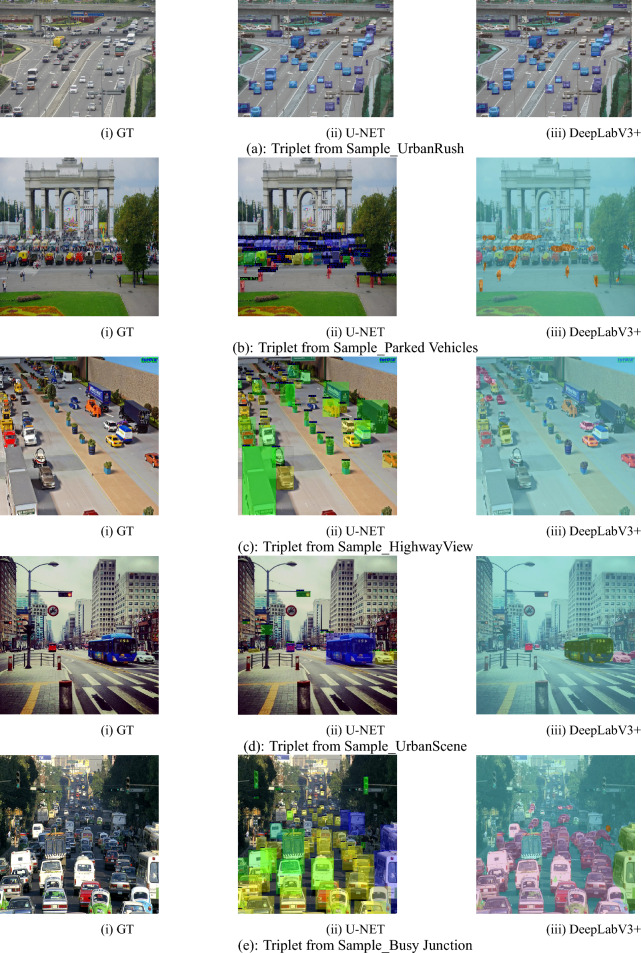
Evaluating segmentation architectures: U-Net vs DeepLabV3+.

It has been attempted to visually distinguish, to a great extent, the changes of the two semantic segmentation models, U-Net and DeepLabV3+, from one another by examining their results for the same scenes. Both models were visually compared by looking at their segmentations with the ground truth.Each set of three images first shows the original photo, then the segmentation results by U-Net, and finally by DeepLabV3+, which helps to visually compare these two methods side-by-side. The differences between the two models are quite evident in various aspects, such as how they define object boundaries, how satisfactorily they distinguish the background, and how well their results conform to the structure of things.Most importantly, their segmentations vary in the way they depict edge details, small objects, and overlapping regions. A person may also notice differences in the smoothness and continuation of the segmented areas when, for example, a dense or cluttered space is present. This visual comparison also helps to highlight how each model performs differently when it comes to handling a variety of scene complexities, lighting conditions, and object sizes. There are certain areas where one model’s output seems smoother or more filled, thereby revealing distinct feature extraction and spatial awareness mechanisms. The triplets as a whole reveal the different segmentation features of U-Net and DeepLabV3+ under different real-world settings. Figure 10 shows the U-NET vs. DeepLabV3+ model comparison.

**Table 8:** Performance analysis of U-Net and DeepLabV3+ Using PSNR and MSE.

Image	U-NET (PSNR)	DeepLabV3+ (PSNR)	U-NET (MSE)	DeepLabV3+ (MSE)
	40.01	38.51	0.000143	0.000556
	41.93	34.75	0.000064	0.000335
	31.31	34.12	0.000739	0.000385
	40.25	32.71	0.000094	0.000536
	37.19	32.82	0.000190	0.000523

We used two widely used image quality metrics, Mean Squared Error (MSE) and Peak Signal-to-Noise Ratio (PSNR), to compare the performance of the U-NET and DeepLabV3+ models. These were acquired by contrasting each model’s segmented output with the initial ground truth image. Higher PSNR values indicate higher quality and indicate how similar the final image is to the original. Instead, MSE indicates the amount of error between the two images; lower values indicate fewer errors.

**Table 9:** Model evaluation using mAP and mIoU metrics.

Image	U-NET (mAP)	DeepLabV3+ (mAP)	U-NET (mIoU)	DeepLabV3+ (mIoU)
	0.461	0.562	0.580	0.760
	0.903	0.950	0.761	0.792
	0.750	0.950	0.654	0.742
	0.949	0.500	0.734	0.725
	0.750	0.614	0.500	0.813

Based on the result, U-NET could be considered as better model in all five example cases. It very frequently delivered higher PSNR and lower MSE values than DeepLabV3+, which means that its result is more similar to the ground-truth. This proves that U-NET has the ability to hold more information and produce more accurate segmentations. All figures/samples as well as the PSNR and MSE values for each model are displayed in Table 8. This table shows the performance of U-NET and DeepLabV3+ models on two common evaluation metrics in segmentation problems: mean Average Precision (mAP) and mean Intersection over Union (mIoU). Mean Average Precision reflects the ability of the model to detect and classify objects correctly at different confidence levels, how good it is at detecting objects.

**Fig. 11:**
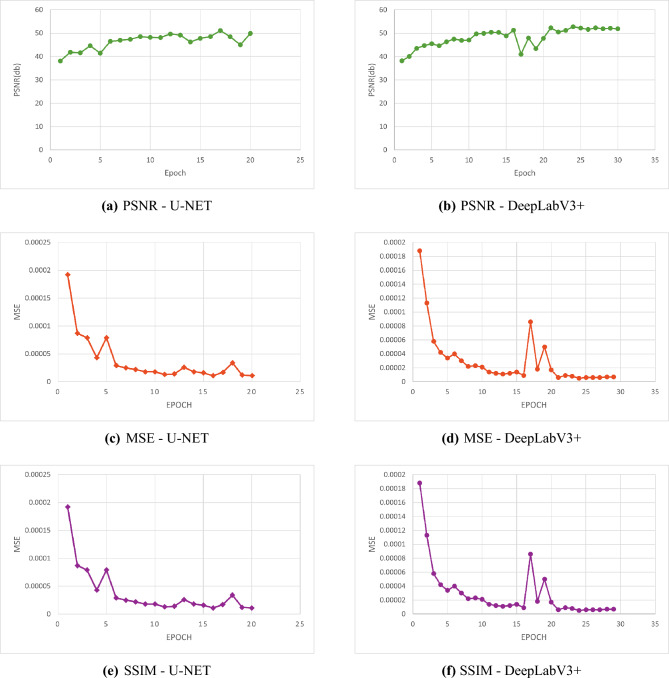
Performance comparison of U-Net and DeepLabV3+ Using PSNR, MSE, and SSIM.

U-NET and DeepLabV3+ models were compared using three image quality metrics: PSNR, MSE, and SSIM. U-NET was trained for 20 epochs, while DeepLabV3+ was trained for 30 epochs. Although U-NET had fewer training epochs, it produced much higher PSNR and SSIM values and had a very much lower MSE, reflecting its better reconstruction performance. Increased PSNR and SSIM indicate improved perceptual quality and structural similarity, whereas decreased MSE indicates less reconstruction error. All these results evidently prove that U-NET is more efficient and better than DeepLabV3+ for this purpose. Graphical comparison of these metrics is shown in Fig. 11. Qualitative segmentation maps are shown to visually compare boundary accuracy, object continuity, and background separation between U-NET and DeepLabV3+. Figure 11 presents comparative bar charts of PSNR, MSE, and SSIM values for both U-NET and DeepLabV3+, providing a clear graphical comparison of reconstruction performance.

Mean Intersection over Union is the metric of average overlap degree of the predicted segmented region with the ground-truth, reflecting the accuracy of the segmented region.Area mIoU higher indicates that the predicted segmented shapes are very close to the actual shapes in the image, which is especially crucial for pixel-level accuracy based applications. As per the experiments, U-NET mostly has higher mIoU values, indicating that it is more accurate and thorough when segmenting than the other methods. DeepLabV3+, although better in mAP for certain scenarios, is inconsistent with regions in others.In general, U-NET shows a better capacity in maintaining object contours and boundaries and is thus more trustable for use in medical imaging, autonomous vehicles, or traffic flow analysis where precise segmentation is required. Table 9.

**Fig. 12:**
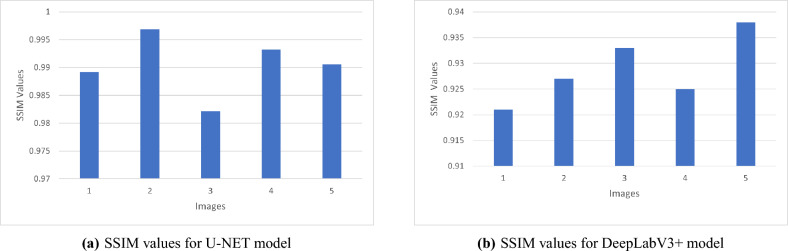
Image quality evaluation: U-Net vs DeepLabV3+ Using SSIM.

It is compared against mIoU (Mean Intersection over Union) and mAP (Mean Average Precision) on three methods.Multi-Scale Attention U-Net with EfficientNetB4 backbone yielded the highest mIoU of 0.813, which is extremely high segmentation accuracy.Mask DINO showed the worst numbers in both metrics, while U-Net-based approach exhibited comparable mAP behavior.These results are summarized in Table 10.

**Table 10:** Performance comparison of methods using mIoU and mAP.

Author(s)	Method	mIoU	mAP
Li et al.^23^	Mask DINO	0.608	0.545
Preetha et al.^24^	Multi-Scale Attention U-Net + EfficientNetB4	0.879	–
Proposed Work	U-NET	0.761	0.949

In this study,the comparison involves PSNR (Peak Signal-to-Noise Ratio), MSE (Mean Squared Error), and SSIM (Structural Similarity Index Measure) over four approaches: SEI (Scale-Equivariant Imaging), LSB (Least Significant Bit), U-Net (U-shaped Convolutional Neural Network), and Bicubic Interpolation. The U-Net method introduced achieves the best reconstruction performance resulting in the highest PSNR (41.93 dB), the lowest MSE (0.000094), and the highest SSIM (0.99688). On the other hand, the SEI method by Cammarasana et al.^25^ has been identified as the least effective method among the tested approaches. The results of these experiments are shown in Table 11. The SSIM bar charts also reflect that the U-Net model generates higher SSIM values than DeepLabV3+ for all five test images, which implies that U-Net is more capable of maintaining the visual quality and structural aspects of the original images. Figure 12 exhibits the corresponding SSIM comparison. In fact, the SSIM values that were shown in Images 2 and 4 for the U-Net model were extremely high so high that, from a perceptual point of view, the reconstructed features looked even more detailed and consistent than the corresponding low-resolution inputs. On the other hand, the DeepLabV3+ model had lower SSIM values for all five test images, which suggests that the model was less capable of preserving the structural details of the original image during segmentation. From these experiments, it seems that U-Net has a higher capacity to preserve fine structural details as well as keep the overall visual quality of the image. As a result, U-Net is very efficient in situations where perceptual similarity and structural accuracy literally mean the most. Hence, our results constitute a definite proof that U-Net is superior to DeepLabV3+ when compared by SSIM. Nevertheless, these observations are intended only as qualitative indicators rather than formal benchmark measurements, and the analysis will be extended in future work.

**Table 11:** Quantitative performance comparison using PSNR, MSE, and SSIM.

Author(s)	Method	PSNR (dB)	MSE	SSIM
Yufei Jiang et al.^26^	Bicubic	30.66	–	0.915
Ramyashree et al.^27^	LSB	26.36	0.375	0.878
Cammarasana et al.^25^	SEI	11.66	0.443	0.078
Proposed method	U-Net	41.93	0.001	0.996

### Limitations

There are several limitations which the proposed super-resolution assisted semantic segmentation framework has to overcome despite the fact that it exhibits strong performance on urban traffic scenes. The experiment was only conducted on a small subset of static images that may not even partially reflect the diversity and complexity of large-scale real-world traffic environments. It has not been explicitly studied whether the framework could generalize well to extreme traffic conditions, like heavy congestion, bad weather, night-time illumination, and severe occlusions. Beside the suggested two-stage pipeline would increase the computational cost because of the super-resolution preprocessing step before segmentation. Although this enhances the visual quality and the segmentation accuracy, it may limit real-time implementation on low-powered embedded platforms that are typically used for autonomous driving and traffic surveillance systems. Moreover, the present work lacks cross-dataset validation and video-based evaluation making the conclusions on robustness and generalizability to changing traffic situations tentative.

## Conclusion and future work

An in-depth semantic segmentation study for traffic scenes shows that resolution enchancement through super-resolution leads to a significant improvement in the segmentation accuracy of deep learning-based models. In this research, two widely used architectures, namely U-Net and DeepLabV3+, were compared on a carefully filtered subset of the COCO dataset with traffic classes. The inclusion of a no-super-resolution control group made it possible to determine that the increases in segmentation accuracy were primarily the result a the enhancement stage, since models using non-enhanced low-resolution images consistently yielded lower mIoU and mAP scores. The results reflect that DeepLabV3+ obtained more accurate segmentation with a best mIoU of 0.761 and best mAP of 0.949, whereas U-Net obtained better image reconstruction quality with a best PSNR of 41.93 dB and lowest MSE of 0.001. Pre-processing images before semantic segmentation enables the models to identify and label traffic objects like vehicles, pedestrians, road signs, and infrastructure more effectively. Furthermore, a corrected resolution of 640 × 640 may serve as a real-world scenario for checking the model’s performance and, simultaneously ,while also providing computational efficiency. This arrangement can be extremely helpful for the implementation of hardware with a limited number of resources, such as an embedded system in an autonomous vehicle. Besides, the model’s reliability under varying visual scenarios is further proven by the performance metrics. DeepLabV3+ is more suitable when we need very accurate segmentation, whereas U-Net can reconstruct the image stably and keep the visual appearance intact. The trade-off between these models clearly shows that our pipeline can be very effective for intelligent transportation systems operating in diverse traffic scenes. Nevertheless, the operational limitations of the proposed method should not be overlooked as well. The precision of segmentation might deteriorate in extreme weather or low visibility situations, for instance, heavy rain, thick fog, night glare, or lack of light, where super-resolution may not be able to rescue the essential structural details entirely. Moreover, making the system work in real-time is another problem since the combination of super-resolution and segmentation increases the computation load significantly. Due to this additional complexity, there is often a delay and device performance limitations in edge devices that are usually employed in intelligent transportation systems. Besides that, the models were tested only on a particular subset of the COCO dataset, which indicates that their performance may vary when they are placed in different settings or with different sensors that are not the parts of the training data. Future research will mainly focus on solving these limitations by means of light architectures, the enhancement of weather-robust features, and generalization of cross-dataset. Further experiments can explore the integration of object tracking, multi-frame video processing, and real-time deployment. The segmentation system designed as a part of this study can be very powerful in enhancing city transport, the perception of autonomous vehicles, and traffic monitoring. Other possibilities for future research are deployment confirmation and regulatory alignment with transport infrastructure organizations.

## Data Availability

The data sets analyzed during the current study are available in curated subset of the Microsoft COCO dataset $\phantom{0}^{16}$.
